# Susceptibility of Select Agents to Predation by Predatory Bacteria

**DOI:** 10.3390/microorganisms3040903

**Published:** 2015-12-02

**Authors:** Riccardo Russo, Richard Chae, Somdatta Mukherjee, Eric J. Singleton, James L. Occi, Daniel E. Kadouri, Nancy D. Connell

**Affiliations:** 1 Department of Medicine and the Center for Emerging Pathogens, Rutgers, New Jersey Medical School, Newark, NJ 07101, USA; E-Mails: russori@njms.rutgers.edu (R.R.); singleej@njms.rtugers.edu (E.J.S.); occijl@njms.rutgers.edu (J.L.O.); 2 Department of Oral Biology, Rutgers School of Dental Medicine, Newark, NJ 07101, USA; E-Mails: rkchae@buffalo.edu (R.C.); smukherjee@fordham.edu (S.M.); kadourde@sdm.rutgers.edu (D.E.K.)

**Keywords:** predatory bacteria, *Bdellovibrio*, *Micavibrio*, Select Agents

## Abstract

Select Agents are microorganisms and toxins considered to be exploitable as biological weapons. Although infections by many Select Agents can be treated by conventional antibiotics, the risk of an emerging or engineered drug resistant strain is of great concern. One group of microorganisms that is showing potential to control drug resistant Gram-negative bacteria are the predatory bacteria from the genera *Bdellovibrio* spp. and *Micavibrio* spp. In this study, we have examined the ability of *Bdellovibrio bacteriovorus* (*B. bacteriovorus*) strain 109J, HD100 and *Micavibrio aeruginosavorus* (*M. aeruginosavorus*) ARL-13 to prey on a variety of Select Agents. Our findings demonstrate that *B. bacteriovorus* and *M. aeruginosavorus* are able to prey efficiently on *Yersinia pestis* and *Burkholderia mallei*. Modest predation was also measured in co-cultures of *B. bacteriovorus* and *Francisella tularensis*. However, neither of the predators showed predation when *Burkholderia pseudomallei* and *Brucella melitensis* were used as prey.

## 1. Introduction

The use of predatory bacteria, such as *Bdellovibrio bacteriovorus* and *Micavibrio aeruginosavorus*, has been recommended as an alternative approach to the development of antibacterial agents [1]. These Gram negative bacteria are obligate predators that prey on a wide range of Gram negative bacteria [2,3]. The predators are smaller than their prey. The length of *Bdellovibrio* spp., for example, is generally stated to be <1 uM, or approximately half as long as *Escherichia coli*; when *Bdellovibrio* emerge from prey, they are generally larger than their planktonic size these changes are correct [4]. Originally recovered from environmental water and soil, the predators are now considered ubiquitous, having now been localized to animal intestines [5,6,7]. *Bdellovibrio* spp. use a single flagellum to propel through the medium until they encounter a prey cell, to which they attach by an unknown mechanism. Over the next 2 h, they move through a series of well-described stages: They enter the periplasmic space, begin to extract nutrients from within the cell, replicate by filamentation and eventually lyse the cell, releasing new predators. By means of this simple life cycle, predator bacteria can clear a dense culture of prey within 12–18 h under laboratory conditions. The concept of developing predatory bacteria for therapeutic use against Gram negative bacterial infections is gaining momentum. Several publications from this and other laboratories have described and cataloged the range of Gram negative bacteria that are attacked by *B. bacteriovorus* and *M. aeruginosavorus*, including human pathogens and multi-drug resistant strains [8,9,10,11,12,13]. One significant group of bacteria that has not yet been tested for susceptibility to predation is the “Select Agents”.

Adding to the problem of infectious disease outbreaks is the resurgence of the threat of biological weapons use, beginning with the release and spread of spores of *Bacillus anthracis* through the United States Postal Service in late 2001 [14]. In the wake of this event, which brought concern over biological weapons to the forefront, US government agencies CDC (United States Center for Disease Control) and USDA (United States Department of Agriculture), in consultation with intelligence agencies, have classified microorganisms on the basis of their perceived likelihood to be used as weapons and limited access as is deemed appropriate.[15]. Select Agents comprise a group of specific biological agents (bacteria, viruses, fungi and toxins) designated by United State Department of Health and Human Services and/or the U.S. Department of Agriculture to have the potential for use or development as a biological weapon [16].

There are eleven “Tier 1” Select Agents, and of these, five are bacteria: *Francisella tularensis*, *Bacillus anthracis*, *Burkholderia mallei*, *B. pseudomallei* and *Yersinia pestis*; these bacterial species are the causative agents, respectively, of tularemia, anthrax, glanders, melioidosis and plague. With the exception of *B. pseudomallei* (melioidosis), these five species cause zoonotic infections and thus have little to no natural reservoir in humans; antibiotic resistance is not common in these strains, although the *Burkholderia* spp. possess high innate resistant to most antibiotics [17,18].

In the years since the dissemination of *Bacillus anthracis* spores through the US mail [14], there have been significant advances in detection and decontamination of biowarfare agents, as well as deeper understanding of pathogenesis of and immunity to these infectious agents [16,19,20]. However, antibiotic resistance remains a potential weakness; the use of intrinsically-resistance species (such as *Burkholderia* spp.), antibiotic-resistant strains in a deliberate or natural outbreak would lead to even more challenging response and mitigation requirements [21,22]. Here, we describe the use of predatory bacteria as an alternative approach to the control of bacterial infections by examining the *in vitro* susceptibility of a series of Gram negative Select Agent bacteria to predation by *Bdellovibrio bacteriovorus* and *Micavibrio aeruginosavorus.*

## 2. Experimental Section

**Bacteria, strains and growth conditions:** The predatory bacteria used in the study were *Micavibrio aeruginosavorus* strain ARL-13 [23], *Bdellovibrio bacteriovorus* HD100 [24] and *Bdellovibrio bacteriovorus* 109J (ATCC 43826), as listed in Table 1. Predatory bacteria were cultured as described previously [8]. In brief, predator stock-lysates were prepared by co-culturing the predators with host cells in diluted nutrient broth (DNB) (1:10 dilution of nutrient broth supplemented with MgCl_2_ 3 mM and 2 mM CaCl_2_). The co-cultures were incubated at 30 °C until the culture cleared. To grow the predators for each predation experiments, 2 mL of predatory bacteria from the stock-lysates were added to 20 mL of DNB containing 2 mL overnight washed host cells (~1–5 × 10^8^ CFU/mL final concentration). *B. bacteriovorus* and *M. aeruginosavorus* co-culture were incubated for 24 and 48 h, respectively. Thereafter, the co-cultures were filtered through a 0.45-µm Millex pore-size filter (Millipore, Billerica, MA, USA) to obtain a final predator concentration of ~1–5 × 10^8^ PFU/mL (harvested predator).

*Brucella mellitensis* (16M), *Burkholderia mallei* (China 5 and China 7), *B. pseudomallei* (Human/Blood/OH/US/1994;), *Francisella tularensis* (Schu 4 and WY96-3418), *Klebsiella pneumoniae* subsp. *pneumoniae* (ATCC 43816) and *Yersinia pestis* (CO92 and 125 Bombay) were grown at 37 °C in broth and agar plates based on the media listed in Table 1.

**Predation analysis:** Predation experiments were conducted as described previously [9]). Five-milliliter co-cultures were prepared by adding 0.5 mL of harvested predator (~1–5 × 10^8^ PFU/mL) to 0.5 mL of washed host cells (~1–5 × 10^9^ CFU/mL) to 4 mL of DNB. Predator-free prey cells were used as control. The culture tubes were placed at 30 °C in a rotatory drum shaker set at 20 rpm. For semi-quantified predation analysis, predation was determined by the change in prey population, measured by the change in culture turbidity at 600 nm (OD_600_) as compared with untreated culture, which does not increase in turbidity in DNB. For a subset of Select Agents that showed turbidity reduction when co-cultured with the predator, a second predatory predation assay was conducted. In this assay, the change in prey population was measured by dilution plating and CFU enumeration. All experiments were conducted at least twice in triplicate.

**Table 1:** Bacterial strains used in the study.

Organism	Strain Name	Origin	Liquid Medium	Agar Medium
*Bdellovibrio bacteriovorus*	109J	ATCC (43826)	N/A ^1^	
*Bdellovibrio bacteriovorus*	HD100	[24]		
*Micavibrio aeruginosavorus*	ARL-13	[23]		
*Brucella mellitensis*	16M—NCTC 10094	BEI Resources (NR-256)	BB	BA
*Burkholderia mallei*	China 5—MM-A, NBL 4	BEI Resources (NR-21)	BHI	BHIA
*Burkholderia mallei*	China 7—NBL 7	BEI Resources (NR-23)	BHI	BHIA
*Burkholderia pseudomallei*	Human/Blood/OH/US/1994	CDC (2000032029)	BHI	BHIA
*Burkholderia pseudomallei*	1710a	BEI Resources (NR-8071)	BHI	BHIA
*Burkholderia pseudomallei*	K96243	BEI Resources (NR-4073)	BHI	BHIA
*Francisella tularensis*	Schu 4	Bacteriology Division USAMRC	CHB	CA
*Francisella tularensis*	WY96-3418	BEI Resources (NR-644)	CHB	CA
*Klebsiella pneumoniae subsp. pneumoniae*	Trevisan	ATCC (43816)	LB	LBA
*Yersinia pestis*	C092	BEI Resources (NR-641)	BHI	BHIA
*Yersinia pestis*	125 Bombay	BEI Resources (NR-20)	BHI	BHIA

^1^ Media is determined by the prey used in each experiment; BB = BBL™ *Brucella* Broth (Becton, Dickinson and Company, Franklin Lakes, NJ, USA, D 211088); BA = *Brucella* Agar (BBL™ *Brucella* Broth plus Agar (Sigma-Aldrich Corporation, St. Louis, MO, USA, A6686)); BEI resources = Biodefense and Emerging Infections Research Resources Repository (*BEI* Resources); BHI = BBL™ Brain Heart Infusion (BD 211059); BHIA = Brain Heart Infusion Agar (BBL™ Brain Heart Infusion plus agar); CA = Chocolate II Agar with hemoglobin and IsoVitalex (BD 221169); CHB = Cysteine Heart Broth (10 g BHI, 10g Proteose Peptone (Sigma F29185), 10 g Dextrose (Sigma D9434), 5 g Sodium Chloride (Sigma S3014), 1 g L-Cysteine (Sigma C7352) in 1 L water); LB = Luria-Bertani Broth (Sigma L3022); LBA = Luria-Bertani Agar (Luria-Bertani Broth + Agar).

**Safety, biohazards and regulatory compliance:** All work with Select Agents was carried out in the Biosafety Level Three Laboratory of the Rutgers New Jersey Medical School Regional Biocontainment Laboratory, located at the International Center for Public Health 225 Warren Street, Newark, NJ 07103. Select Agent Registration Number: C20140325-1569, effective 25 March 2014, expires 25 March 2017. Only qualified users, as determined by the Rutgers Institutional Biosafety Committee according to the latest federal guidelines, may enter. All protocols are reviewed by the Institutional Biosafety Committee for biosafety, biosecurity and dual use compliance. The implementation of each working protocol is accompanied by a risk assessment and evaluated before initiation by an internal protocol committee.

## 3. Results and Discussion

The spread of antibiotic resistant infectious disease agents is one of the world’s greatest contemporary crises. Over the past several decades, emerging and reemerging infectious diseases have had a grave impact on society and economic stability across the globe. The combination of lives lost (> 13 million per year) and the cost of outbreaks (the recent Ebola outbreak approached $32 billion) [25] is exacerbated by the increase in multidrug resistant strains of bacteria, viruses and fungi. While the past two decades of biomedical research have seen greatly expanded understanding of pathogenesis and immunology, novel antimicrobial development has been slow, and very few new drugs have entered the pipeline [26].

In this setting of the increasing threat of antibiotic resistant bacteria, predatory bacteria represent an alternative approach to traditional antibiotics, which target essential cellular functions such as protein, DNA, RNA and cell wall synthesis. Predatory bacteria attack and destroy Gram negative bacteria irrespective of growth state or antibiotic resistance status, and have been under investigation for use against human pathogens [8,27,28]. Here, we demonstrate that *Bdellovibrio bacteriovorus* strains 109J and HD100 and *Micavibrio aeruginosavorus* ARL-13 can attack certain species of Select Agent bacteria.

To examine the host range and effectiveness of *B. bacteriovorus* and *M. aeruginosavorus* to attack and reduce CFUs of bacterial Select Agents, *Y. pestis*, *F. tularensis*, *B. mallei*, *B. pseudomallei* and *B. melitensis* were cultured and incubated in the presence of the predator strains. Two virulent strains of *Y. pestis* showed approximately 50% reduction (range: 49%–56%) in turbidity at 48 h when co-cultured with two strains of *B. bacteriovorus*; reduction of these strains by *M. aeruginosavorus* ARL-13 was 42% and 44% (Table 2). The survival of these *Y. pestis* strains when measured by CFU enumeration showed more variation (Table 3): *B. bacteriovorus* 109J demonstrated a CFU log reduction of 4, whereas *B. bacteriovorus* HD100 and *M. aeruginosavorus* ARL-13 were only 1.5 and 1.9 log, respectively.

Two *B. mallei* strains were tested for susceptibility to the three predator strains. The *B. bacteriovorus* strains 109J and HD100 both showed the ability to reduce turbidity by 72%–80%, whereas *M. aeruginosavorus* ARL-13 reduced turbidity during co-culture by only 6% (*B. mallei* China 7) and 27% (*B. mallei* China 5). The CFU reduction of both *B. mallei* strains was quite effective, between 4.1 and 5.3 logs over 48 h. As with the turbidity experiments, *M. aeruginosavorus* ARL-13 was less effective: Reduction of *B. mallei* China 5 and China 7 was 0.8 and 1.6 logs, respectively.

Finally, *F. tularensis* Wyoming 96 and SHU4 were both attacked, albeit weakly, by both *B. bacteriovorus* strains: *B. bacteriovorus 109J* by 21% and 29% and *B. bacteriovorus* HD100 by 7% and 9%, respectively; neither *F. tularensis* strains were at all susceptible to predation by *M. aeruginosavorus* ARL-13.

Two other Select Agent species were investigated, *B. pseudomallei* and *B. melitensis* (the latter is a non-tier 1 Select Agent), and neither was susceptible to attack by either predator species. This result was not entirely unexpected; Kadouri and colleagues, and other groups have performed extensive analysis of host range of the three predator species studied here, and identified other Gram negative species have been refractory to predation [8,11]. The mechanisms governing susceptibility to predation are an active area of investigation. For example, a recent study described sequential the cues provided by both the prey and predator required for cell cycle progress [28]. Examination of conditions that affect predation [29,30] and the observation that breach in host specificity can occur over time [31] suggest that experimental manipulation is possible to expand the host range of the predators. Interestingly, the difference in susceptibility to predation shown by *B. mallei vs. B. pseudomallei* might be exploited in genetic approaches to understand further the nature of predator susceptibility.

**Table 2:** Reduction (%) in prey cell turbidity following predation.

Predator			
	*B. bacteriovorus* 109J	*B. bacteriovorus* HD100	*M. aeruginosavorus* ARL-13
**Prey** ^1^			
*Y. pestis* plague Bombay	+ ^2^	+	+
	(45 ± 13%)	(39 ± 7%)	(8 ± 2%)
	(55 ± 15%) *	(54 ± 6%) *	(42 ± 17%) *
*Y. pestis* NR-641 CO92	+	+	+
	(39 ± 14%)	(16 ± 5%)	(17± 10%)
	(56± 14%) *	(49 ± 17%) *	(44 ± 15%) *
*B. mallei* NR-21 China 5	+	+	+
	(66 ± 4%)	(65 ± 3%)	(7 ± 2%)
	(72 ± 6%) *	(66 ± 6%) *	(27±5%) *
*B. mallei* NR-21 China 7	+	+	+
	(66 ± 9%)	(67 ± 5%)	(5 ± 2%)
	(80 ± 5%) *	(69 ± 2%) *	(6 ±2%) *
*B. pseudomallei* NR-8071 1710a	− ^3^	−	−
*B. pseudomallei* NR-4073 K96243	−	−	−
*B. pseudomallei* OH	−	−	−
*F. tularensis* NR-644. WY96-3418	+	+	−
	(10 ± 3%)	(6 ± 1%)	
	(21 ± 3%) *	(7 ± 0.5%) *	
*F. tularensis* SCHU 4	+	+	−
	(18 ± 4%)	(2 ± 1%)	
	(29 ± 4%) *	(9 ± 2%) *	
*B. melitensis* NR-256 16M	−	−	−

^1^ Co-cultures were prepared by adding prey cells to harvested predator cells or predator free control. Data represent the % reduction in culture turbidity as compared to the predator free control, following 24 and 48 h (*) of incubation. Each experiment was conducted in triplicate. Values represent mean and standard error; ^2^ (+) Positive predation (reduction in culture turbidity); ^3^ (−) Negative predation (no reduction in culture turbidity).

**Table 3:** Change (log10 reduction) in prey cell viability.

Predator			
	*B. bacteriovorus* 109J	*B. bacteriovorus* HD100	*M. aeruginosavorus* ARL-13
**Prey** ^1^			
*Y. pestis* NR-641 CO92	4 ± 0.3	1.5 + 0.5	1.9 + 0.2
*B. mallei* NR-21 China 5	4.6 ± 0.5	4.1 ± 0.4	0.8 ± 0.2
*B. mallei* NR-21 China 7	5.3 ± 0.5	4.7 ± 0.5	1.6 ± 1.1

^1^ Select Agents were co-cultured by adding prey cells to harvested predator or predator free control. Values represent the Log_10_ reduction measured following 48 h of incubation as compared to the predator free control. Each experiment was conducted in triplicate. Values represent mean and standard error.

The mechanism(s) of host specificity and its effect differential susceptibility of strains within the same species is well-documented [2,8,11,12,13,31,32] but not understood. The specifics of attachment to the outer leaf of the outer membrane of the prey are not known but may be associated with unidentified differences in prey surface structures. Other steps in predation—penetration, replication, and escape—may also play a role in strain specificity. In addition, there may be effects from substances, such as toxins, or other molecules, secreted by prey calls that might inhibit predation. For example, in a publication from this laboratory [29], inhibition of predation was demonstrated to result from acidification of the medium by fermentation/catabolism of carbohydrates; the *Micavibrio* species studied here is acutely sensitive to low pH. Strain specificity in bacterial predation is an area of vigorous investigation.

## 4. Conclusions

Predatory bacteria (*B. bacteriovorus* and *M. aeruginosavorus)* are able to pray on a subset of the Tier 1 Select Agents deemed potential biological weapons. *Yersinia pestis* and *Burkholderia mallei* were susceptible to predation, whereas *Francisella tularensis* was less vulnerable; the strains of *Burkholderia pseudomallei* and *Brucella melitensis* studied here were entirely resistant to predation. None of the species of prey bacteria subjected to predation resistance studies to date appears to be capable of developing resistance to predator bacteria [33]. Once their clinical utility is demonstrated, these predators may be a useful alternative therapeutic or may serve an ancillary role for current therapies by assisting in reducing the bacterial population in Gram negative infections [1]. Recent work addressed some of the safety concerns associated with the utilization as a therapeutic methodology [34]. The study indicated that exposure of mice intranasal or intravenous inoculation to high levels of *B. bacteriovorus* and *M. aeruginosavorus* led to no reduction in mouse viability, and quick clearance of the predator bacteria from the lungs and bloodstream. Finally, predators have been shown to be very effective in degrading biofilms *in vitro*, and thus may be effective in industrial and clinical biofilm-like settings [30].
